# Capacity and architecture of emotional face-ensemble coding

**DOI:** 10.1167/jov.25.6.10

**Published:** 2025-05-27

**Authors:** Daniel Fitousi

**Affiliations:** 1 Department of Psychology, Ariel University, Ariel, Israel

**Keywords:** ensemble coding, extraction of summary statistics, capacity coefficient, emotion recognition

## Abstract

The ability to process emotion in ensembles of faces is essential for social functioning and survival. This study investigated the efficiency and underlying architecture of this ability in two contrasting tasks: (a) extracting the mean emotion from a set of faces, and (b) visually searching for a single, redundant-target face within an ensemble. I asked whether these tasks rely on similar or distinct processing mechanisms. To address this, I applied the *capacity coefficient*—a rigorous measure based on the entire response time distribution. In Experiment 1, participants judged the average emotion of face ensembles. In Experiments 2 and 3, participants searched for a predefined emotional target among multiple faces. In both tasks, *workload* was manipulated by varying the number of faces in the display. Results revealed that ensemble averaging is a super-capacity process that improves with increased workload, while visual search is capacity-limited and impaired by greater workload. These findings suggest that averaging is a preattentive process supported by a coactive, summative architecture, whereas visual search is attention-dependent and governed by a serial or parallel architecture with inhibitory interactions between display items.

## Introduction

The present effort addresses a practical and theoretical question concerning the human ability of detecting, recognizing, and averaging emotions from ensembles of faces (Haberman, Lee, & Whitney, 2015; Haberman & Whitney, 2007; Hansen & Hansen, 1988; Öhman, Flykt, & Esteves, 2001; Son et al., 2023; Yang, Yoon, Chong, & Oh, 2013). Imagine that you are facing a crowd of people who are marching toward you in a threatening way, and you should decide immediately whether to stay or run away. To save your life, you must estimate the emotional expression on those faces. If, on average, the faces in the crowd are angry, you are probably at risk. If, on average, the faces in the crowd are happy, you are safe. Recent studies have shown that humans can briefly and accurately estimate the average emotion expression of a set of faces (Haberman, Harp, & Whitney, 2009; Haberman & Whitney, 2007; Sweeny, Grabowecky, Suzuki, & Paller, 2009), as well as other facial aspects, such as identity or gender (Leib et al., 2014; Whitney & Yamanashi Leib, 2018). Earlier studies have documented similar apparently effortless averaging abilities with nonfacial attributes such as size (Chong & Treisman, 2003).

However, averaging is not the only cognitive operation that can be performed by an observer when presented with face ensembles. For example, observers may engage in a visual-search (Hansen & Hansen, 1988; VanRullen, 2006) or a target-detection (Fitousi, 2021c; Miller, 1982) task whereby they look for a predefined target (e.g., an angry face). These tasks are consequential for survival to the same degree, or even more than averaging. Consider again the example of a crowd of people who are marching toward you. You might want to detect a single angry face in that crowd rather than compute the average in order to prepare for a fight-or-flight response (Hansen & Hansen, 1988). While research on processing of face ensembles in recent years has focused mainly on averaging (Haberman, Harp, & Whitney, 2009; Haberman & Whitney, 2007; Sweeny et al., 2009), there has been an earlier and not-less important line of research on visual search with face ensembles (Hansen & Hansen, 1988; Hershler & Hochstein, 2005; Robitaille & Harris, 2011; Suzuki & Cavanagh, 1995; Won & Jiang, 2013). Averaging and visual-search operations are quite different from each other but, when studied together, may provide valuable insights into the underlying mechanisms. In averaging, observers are asked to ignore the individual items and extract the gist of the set, whereas in visual search, the opposite is true—observers are required to focus on the item-level and ignore the overall context. Another marked difference is that averaging likely requires *exhaustive processing* of all or most (Baek & Chong, 2020) items in the display, whereas visual search can be accomplished by adopting a so-called *self-terminating* stopping rule (Sternberg, 1966), according to which processing halts once a predefined target is found.

Here I address the question of whether averaging and visual-search tasks with face ensembles are governed by same or different processing strategies. In particular, the goal is to investigate the *architectures* (i.e., serial/parallel), *stopping rules* (i.e., self-terminating/exhaustive), and *capacity* requirements (i.e., limited-, unlimited-, super-capacity) involved in the two tasks. I therefore harness comparable stimuli and computational frameworks, but with different instructions (averaging vs. visual search). Averaging and visual search may or may not be sustained by the same processing mechanisms. This is an empirical question the present study aims to answer. To address this question, I apply the redundant-target task (Miller, 1982), along with a powerful measure of efficiency known as the *capacity coefficient* (Townsend & Nozawa, 1995; Townsend & Wenger, 2004b). The latter is a response time (RT)–based measure on the entire RT distributions. These tools allow me to test several theoretically plausible mechanisms. For example, it may be the case that averaging is an *unlimited-capacity* process that requires minimal investment of attention and instantiated in a parallel-exhaustive architecture, whereas target detection is a *limited-capacity* process, sustained by a serial-exhaustive architecture. Questions about capacity and architecture can also provide more insights into the attentional requirements of these two tasks. In the next sections, I review the central findings and ideas.

## Does averaging of emotion require attention?

It is tempting to answer “no” because it would be otherwise difficult to explain how observers accomplish high levels of accuracy and rapid responses when they extract the average emotion of faces in an ensemble (Haberman & Whitney, 2007; Leib et al., 2014). In that case, one would like to argue that faces are processed preattentively and therefore require minimal effort. This may explain how people can process many faces at once. If faces demanded attention, then ensemble processing should have been a slow and error-prone process, which it appears not to be case, and indeed, several researchers have argued that statistical averaging of simple and complex objects does not require attention (Alvarez & Oliva, 2009). Averaging of emotion can survive crowding (Fischer & Whitney, 2011). It can be performed without being part of task demand (Haberman, Harp, & Whitney, 2009). In addition, the accuracy of averaging performance does not depend on set size (Chong & Treisman, 2003). Moreover, several studies (Cha & Chong, 2018; Cho, Im, Yoon, Joo, & Chong, 2023; Robitaille & Harris, 2011) have shown that ensemble processing speeds up rather than slows down as more items are added to the display. The improvement in efficiency as a function of increasing workload resembles gestalt phenomena, whereby the sum is greater than its parts (Algom & Fitousi, 2016). In terms of capacity, it can be argued that such a process is characterized by super-capacity processing (Townsend & Nozawa, 1995), one that does not require effort or attention.

However, there are other findings that do not align well with the view that averaging is preattentive. Take, for example, the finding by Haberman and Whitney (2010) that observers represented more precisely the local mean of a set of emotional faces rather than its global mean. This entails that observers can willingly downweight or discount items that are outlying from the central tendency of the distribution. Li, Herce Castañón, Solomon, Vandormael, and Summerfield (2017) have shown that this so-called “robust averaging” (De Gardelle & Summerfield, 2011) is beneficial because it provides a shield against the influence of noise. Utilization of such a strategy likely requires attention to individual items, and observers use it to reduce processing load or capacity demands. Haberman and Whitney (2010) noted that “ensemble expression perception is fast, automatic, implicit, and relatively insensitive to outliers. However, we cannot conclude that attention plays no role. Indeed, recognizing any face—even a single face—may involve attention” (p. 1837). Moreover, most studies employ a high degree of item regularity, which enables participants to sample only a portion of the items in the set (Myczek & Simons, 2008) and thus to maintain an unlimited-capacity processing. However, manipulations that affected capacity did show an influence on the efficiency of averaging. Minimizing item regularity resulted in decreased averaging efficiency (Marchant, Simons, & de Fockert, 2013). In yet another study, Elias, Padama, and Sweeny (2018) have shown that dual-task disruption of attention eliminated averaging of emotions. Similar findings obtained with nonface stimuli (Jackson-Nielsen, Cohen, & Pitts, 2017). Attarha and colleagues (Attarha & Moore, 2015; Attarha, Moore, & Vecera, 2016) have deployed the *simultaneous-sequential* paradigm (Shiffrin & Gardner, 1972). They found that averaging performance in simultaneous presentation mode was better than performance in sequential presentation mode. Their findings are consistent with the view that averaging is a limited-capacity process. These limitations could not be attributed to crowding, low-distractor discriminability, or a limited-capacity comparison process.

## Does visual-search of emotion require attention?

One of the first studies to apply a visual-search task with emotional faces was conducted by Hansen and Hansen (1988). They asked participants to search for an angry face among neutral or happy faces. Search slopes remained constant irrespective of the number of distractors, a result that was interpreted as “an anger superiority effect.” The authors claimed that angry faces are processed preattentively according to a parallel search. Subsequent visual-search studies amassed evidence for resource-free, automatic, and parallel processing of faces irrespective of emotional expression (Lavie, Ro, & Russell, 2003). Several studies (Brown, Huey, & Findlay, 1997; Hershler & Hochstein, 2005; Kuehn & Jolicoeur, 1994; Nothdurft, 1993; Purcell, Stewart, & Skov, 1996) have deployed visual search tasks with displays of emotionally neutral faces. These studies recorded performance (RTs, accuracy) as a function of display set size under the assumption that an increasing search slope should indicate serial (and therefore attention-demanding) processing, whereas a zero slope should entail parallel (and therefore preattentive) processing (Treisman & Gelade, 1980). Capitalizing on this logic, some of these studies reported parallel processing with faces (Hershler & Hochstein, 2005), whereas others (Brown, Huey, & Findlay, 1997) argued for serial processing. VanRullen (2006), for example, showed that the zero-slope (parallel processing) effect can be reproduced with objects as well, a finding that greatly undermines the claims for uniqueness of faces. Moreover, by controlling the stimuli for low-level aspects, through manipulation of inversion or Fourier transformation, VanRullen could eliminate the parallel processing pattern. However, the logic sustaining the search slope methodology has been shown to be inappropriate (Algom, Eidels, Hawkins, Jefferson, & Townsend, 2015; Townsend, 1971). Take, for example, the common idea that serial processing is marked by a positive slope. This exact pattern can be mimicked by a parallel system with limited capacity (Townsend, 1990; Townsend & Wenger, 2004a). Therefore, the conclusions drawn from the search slope methodology are dubious (see also Fitousi, 2021c), and consequently, other, more appropriate methodologies are needed to investigate this issue.

Won and Jiang (2013) were the first to test hypotheses regarding the attention limitations of ensemble processing using a methodology that bears close affinity with the one deployed here. Their experimental approach is not subjected to the critical weaknesses of the search slope methodology (Algom et al., 2015; Townsend, 1971). They have used a speeded discrimination task with happy and angry faces to measure the gain in multiple face displays. Specifically, by comparing performance in single-face and multiple-face displays, they documented improved performance. This redundancy gain was interpreted as supporting a parallel processing architecture (Raab, 1962). The present study employed a similar experimental task but also harnessed a powerful complementary RT measure on the entire distributions that can speak directly to the issue of processing capacity—the *capacity coefficient* (Townsend & Nozawa, 1995; Townsend & Wenger, 2004b). These tools were applied to both averaging (Experiment 1) and target-detection tasks (Experiments 2 and 3).

## Redundancy gains and redundancy losses

The present study applies the same RT-based tools to both the averaging and visual-search tasks. This affords a common yardstick for measurement and assessment. The first tool is called *redundancy gains* and is derived from performance in the *redundant-target* paradigm (Miller, 1982; Raab, 1962; Townsend & Wenger, 2004b; Fitousi, 2015, Fitousi, 2021c). In the categorization version of this procedure, observers are presented with displays of either a single- or four-target faces (hence the nomenclature “redundant target”). As already noted, this design was first deployed by Won and Jiang (2013). Participants are asked to categorize the emotional expression of the target(s) by pressing one of two buttons (happy or angry). The question of interest is whether participants benefit from the redundant displays that present multiple-face compared to single-target displays. To test this question, researchers compare performance in single and multiple targets by computing the difference in performance between the fastest of the single-target condition and the multiple-target displays (Houpt, Townsend, & Donkin, 2014). This quantity is dubbed redundancy gain:

(1) $$RTgain=RT_{single-target}-RT_{ensemble}$$

A redundancy gain significantly greater than zero indicates that performance benefited from the redundancy of faces in the display. A redundancy loss would suggest that performance was hindered by redundancy. No redundancy gain would entail that redundancy neither facilitated nor hindered performance. A comparable measure exists for accuracy. The expected finding in this paradigm is that RTs would be faster in multiple-face displays compared to single-target displays (Colonius & Diederich, 2004, Colonius & Diederich, 2020; Diederich & Colonius, 1991). The origins of redundancy gains/losses for both self-terminating and exhaustive processing have been studied extensively (Colonius & Diederich, 2004; Miller, 1982). Various processing models have been proposed (Townsend & Wenger, 2004b; Grice, Canham, & Boroughs, 1984; Colonius & Vorberg, 1994; Colonius, 1990). These models take into consideration architectural aspects (i.e., serial, parallel, and coactive), along with capacity characteristics (i.e., limited-, unlimited-, and super-capacity) and stopping rule aspects (i.e., self-terminating, exhaustive). Redundancy gains are expected if processing is held in a parallel unlimited-capacity system according to a self-terminating (minimum time) stopping rule (Miller, 1982). In that case, redundancy gains emerge due to statistical facilitation (Raab, 1962). Another possibility is a coactive system that produces super-capacity. Such a system is expected to produce large redundancy gains. In contrast, in a limited-capacity system, or in a serial exhaustive system, adding more items to the display can hinder rather than assist performance.

One novelty of the present effort is combining the redundant-target design and its attendant theoretical tools with a statistical averaging task (see Experiment 1). It is likely that the operative stopping rule in the averaging task is exhaustive because extraction of summary statistics requires the processing of all or most items in the set. Elaborate explanations on these measures and models are given in the next section because these models are best cast within the framework of the capacity coefficient measure to which I turn next.

## The capacity coefficient

A seminal paper by Townsend and Ashby (1978) presented major conceptual and methodological advancements in the measurement of *processing capacity*. These authors developed various quantitative measures on response-time distributions that adequately capture the meaning of capacity as the amount of *energy* exerted or *effort* invested in a task (Kahneman, 1973) and the impact of increasing workload on this quantity. A central measure of efficiency or capacity proposed by Townsend and Ashby (1978), and in subsequent efforts (Townsend & Wenger, 2004b; Townsend & Ashby, 1983; Townsend & Nozawa, 1995; Wenger & Gibson, 2004; Fitousi & Algom, 2018, Fitousi & Algom, 2020; Fitousi & Wenger, 2011, Fitousi & Wenger, 2013; Fitousi, 2015, Fitousi, 2023), is the hazard function *h*(*t*), which gives the instantaneous intensity with which the system can process an input under a certain load. In particular, the hazard function gives the conditional probability of completing processing in the next instant of time, given that processing has not completed yet. Formally, the hazard function can be written as

(2) $$\;h\left(t\right)=\lim_{\Delta t\to \;0}\frac{P(t\leq T\leq t+\Delta t|T\geq t)}{\Delta t}=\frac{f(t)}{S(t)}$$

where *S*(*t*) = *P*(*RT* ⩾ *t*) is the survivor function, and *f*(*t*) is the probability density function (pdf). The integrated hazard function:

(3) $$H\left(t\right)=\int_{0}^{t}h\left(t^{'}\right)\;dt^{'}$$

provides the cumulative value to time *t* of the hazard function. The identity *H*(*t*) = −ln [*S*(*t*)] is well known and greatly assists in computation. Townsend and Wenger (2004b) note that “the integrated hazard function is a slightly coarser but probably much more stable measure of capacity than is the more microscopic *h*(*t*), where *h*(*t*) is analogous to power and *H*(*t*) to energy or work done” (p. 1017). The integrated hazard function is therefore a central measure of efficiency in response-time tasks (Fitousi & Wenger, 2011).

A second key idea in research on capacity concerns the influence of workload on efficiency of processing (Townsend & Ashby 1978, Townsend & Ashby 1983; Townsend & Wenger 2004b). In particular, increasing the number of items-to-be-processed in the display can harm, facilitate, or leave performance unaffected. The *capacity coefficient* (Townsend & Wenger, 2004b) is a response time–based measure specifically designed to gauge the influence of workload on processing efficiency. The measure deploys the integrated hazard function and compares performance in displays that present all the targets at once to the hypothetical case in which all targets are processed in parallel and with no difference in speed, whether they are alone or together (see discussion in Luce, 1986, of this condition). The latter is deduced from performance with the single-target displays. There are two versions of the capacity coefficient. The first measure is designed to assess efficiency in disjunctive (OR) tasks that can be accomplished by adopting a self-terminating (minimum-time) stopping rule. In such tasks, processing can halt after finding the target, without the need to proceed and process the other elements in the display:

(4) $$C_{OR}\left(t\right)=\frac{H_{E}}{\left[\;\sum_{i=1}^{N}H_{S_{i}}\right]\;}$$

where *H*_*E*_ is the integrated hazard function for performance with face-ensemble displays, and $H_{s_{i}}$ are the integrated hazard function for performance with the single-target displays. More technical details on this coefficient can be found in Appendix A.

The second measure is a conjunctive (AND) measure. In these tasks, processing is exhaustive (Sternberg, 1966). This requires a different intensity function than the integrated hazard function. To this end, Townsend and Wenger (2004b) have proposed an analogous measure—the integrated reverse hazard function *K*(*t*) (Chechile, 2011). This function gives the “conditional probability density that processing completed in just the last instant, given that it completes at or before *t*” (p. 1020). The reverse hazard function is written as

(5) $$k\left(t\right)=\frac{f(t)}{F(t)}$$

and the integrated reversed hazard

(6) $$K\left(t\right)=\int_{0}^{t}k\left(t^{'}\right)\;dt^{'}$$

the identity *K*(*t*) = ln *F*(*t*) (Chechile, 2011) greatly simplifies computations. The AND capacity coefficient is then defined as

(7) $$C_{AND}\left(t\right)=\frac{\left[\;\sum_{i=1}^{N}K_{S_{i}}\right]\;}{K_{E}}$$

where *K*_*E*_ is the integrated reverse hazard function for performance with the face ensemble, and $K_{S_{i}}$ are the integrated reverse hazard functions for performance with the single-target displays. More technical details on this coefficient can be found in Appendix A. The task of averaging (Experiment 1) is likely performed by an exhaustive processing of the display and therefore necessitates the application of the AND capacity coefficient. Simulations (Baek & Chong, 2020) have shown that exhaustive processing is needed for correct averaging with displays of four or fewer items, which is the case in the present study. In contrast, the task of target detection (Experiments 2a, 2b, 3a, 3b) can be accomplished by adopting a self-terminating (minimum time) stopping rule (Miller, 1982; Raab, 1962), and the disjunctive OR capacity coefficient is the appropriate one.

The interpretation of the capacity coefficient for both the OR and AND measure is based on the comparison of performance to an unlimited-capacity independent processing (UCIP) model, which predicts a *C*(*t*) = 1 (Townsend & Wenger, 2004b). The patterns by which performance deviates from this value can inform us on various types of processing capacity. If *C*(*t*) > 1, performance is super-capacity, meaning that ensemble representation of faces facilitates the perception of its individual components. In this case, the channels are dependent on each other, either due to a coactive architecture or due to positive correlations between independent channels (Eidels, Houpt, Altieri, Pei, & Townsend, 2011; Fitousi & Algom, 2018). In any event, super-capacity means that the signals from individual faces interact with each other. If *C*(*t*) < 1, then capacity is limited, meaning that ensemble representation of faces hinders performance with each individual component presented alone.

Townsend (Townsend & Nozawa, 1995; Townsend & Ashby, 1983) provided substantial formal and empirical evidence that aspects of a system’s capacity (limited, unlimited, and super) are independent from characteristics of architecture (serial, parallel) and the stopping rule (exhaustive, self-terminating). But, there are some cases in which architecture and the stopping rule can predict capacity. For example, a parallel system with positive interactions between channels often results in super-capacity, whereas a parallel system with negative interactions often culminates in limited-capacity processing (Eidels et al., 2011; Fitousi & Algom, 2018). Another example is exhaustive serial systems, which are expected to be of limited capacity (Townsend & Nozawa, 1997). Thus, results from the capacity coefficient can provide insights into the underlying architectures and stopping rules (Fitousi, 2019, Fitousi, 2021c).

## Candidate architectures

The processing of face ensembles can be performed according to at least three candidate strategies. The first is a *serial* model in which faces are processed one after the other. In the averaging task, this entails a serial-exhaustive system because information from all faces in the set should be considered to extract the required summary statistics (e.g., average). In the target-detection task, the candidate model is a serial self-terminating architecture because the decision is based on the first face detected. In both cases, no redundancy gains are predicted, and the capacity coefficient is expected to be smaller than 1 [*C*_*and*_(*t*) < 1, *C*_*or*_(*t*) < 1], indicating limited capacity.

The second theoretical possibility is a *parallel* system. The prediction for the averaging task is an exhaustive-parallel system because all items in the display should be processed to compute the average. The channels in this system may incorporate (a) no cross-channel correlation, (b) positive (facilitation) correlation, or (c) negative (inhibitory) correlation. In case (a), we can predict no redundancy gains because there is no statistical facilitation (Raab, 1962) and unlimited capacity (*C*_*and*_(*t*) = 1). In case (b), redundancy gains and super-capacity (*C*_*and*_(*t*) > 1) are expected. In case (c), redundancy losses and limited capacity (*C*_*and*_(*t*) < 1) are predicted. Negative interactions are consistent with the possibility of crowding (Bouma, 1970) or suppression (Desimone & Duncan 1995). According to the latter suppression model of attention, competition between the neuronal activation of stimuli in the visual cortex leads to sensory inhibition by various areas of the visual cortex, including V2, V4, MT, and MST. In the present case, it is predicted that faces in multiple-item displays compete for neuronal representation and therefore suppress each other. The predictions for the target-detection task are similar, but with the likelihood that processing is a self-terminating process. Thus, in case (a), small redundancy gains are expected due to statistical facilitation (Raab, 1962), but capacity should be unlimited (*C*_*or*_(*t*) = 1). In case (b), large redundancy gains are expected along with super-capacity (*C*_*or*_(*t*) > 1). In case (c), large redundancy losses are predicted accompanied by limited capacity (*C*_*or*_(*t*) < 1).

The third model is a *coactive* system (Miller, 1982; Townsend & Nozawa, 1995) in which evidence from each face is accumulated into an integration node, which then sums up activation from all faces (channels). Response is emitted when activation in the integration node breaks a given threshold. In the case of averaging, the threshold might be set according to the standard of comparison. Such a system has been recently implemented by Utochkin, Choi, and Chong (2023) in a two-layer neuronal network with a simple feature layer and a pooling layer. Ensemble representations in this model are conceived as population responses in the pooling layer, which can decode various statistical properties from population responses. The coactive model predicts super-capacity (*C*_*and*_ > 1). Simulations (see Appendix B) of redundancy gains in a simple AND model show that these emerge only when super-capacity exceeds a certain level. All of these predictions are tested in three experiments. Experiment 1 deploys an averaging task, while Experiments 2 and 3 administer a redundant-target task.

## Experiment 1

This experiment implements a speeded averaging task (Haberman & Whitney, 2007) that is embedded within a redundant-target design (Won & Jiang, 2013). Participants are presented with either a single-target face or four-face ensembles and asked to decided whether the average emotional expression is larger or smaller than that of a standard. This design affords the measurement of redundancy gains/losses and the computation of the conjunctive AND capacity coefficient. A critical issue in the processing of face ensembles concerns the type of emotional expression conveyed by the faces. Faces expressing negative emotions (e.g., anger) may require less attention than faces expressing neutral or positive emotions, maybe due to their survival value (Eimer & Holmes, 2007). Several studies (Horstmann & Bauland, 2006; Hansen & Hansen, 1988; Öhman, Flykt, & Esteves, 2001) have documented “an anger superiority effect” by which angry faces are detected more efficiently than happy or neutral faces in a crowd of faces. Moreover, there is evidence that the type of emotion can affect the averaging operation (Ji & Pourtois, 2018; Ji, Pourtois, & Sweeny, 2020). To address this issue, the present study administrated displays with happy or angry faces to examine the impact of emotion on the capacity and architecture of ensemble face processing.

### Methods

#### Participants

Fifty participants took part in this experiment (mean age = 26.3, sd = 2.3, F = 34, M = 16). Participants were recruited from Ariel University pool of participants and compensated with a course credit. The study was performed in accordance with the ethical standards as laid down in the 1964 Declaration of Helsinki. The experiments reported here received the approval of the Ethics Committee of Ariel University (AU-SOC-DF-20230205). All participants gave their informed consent.

#### Stimuli and apparatus

**Figure 1.**
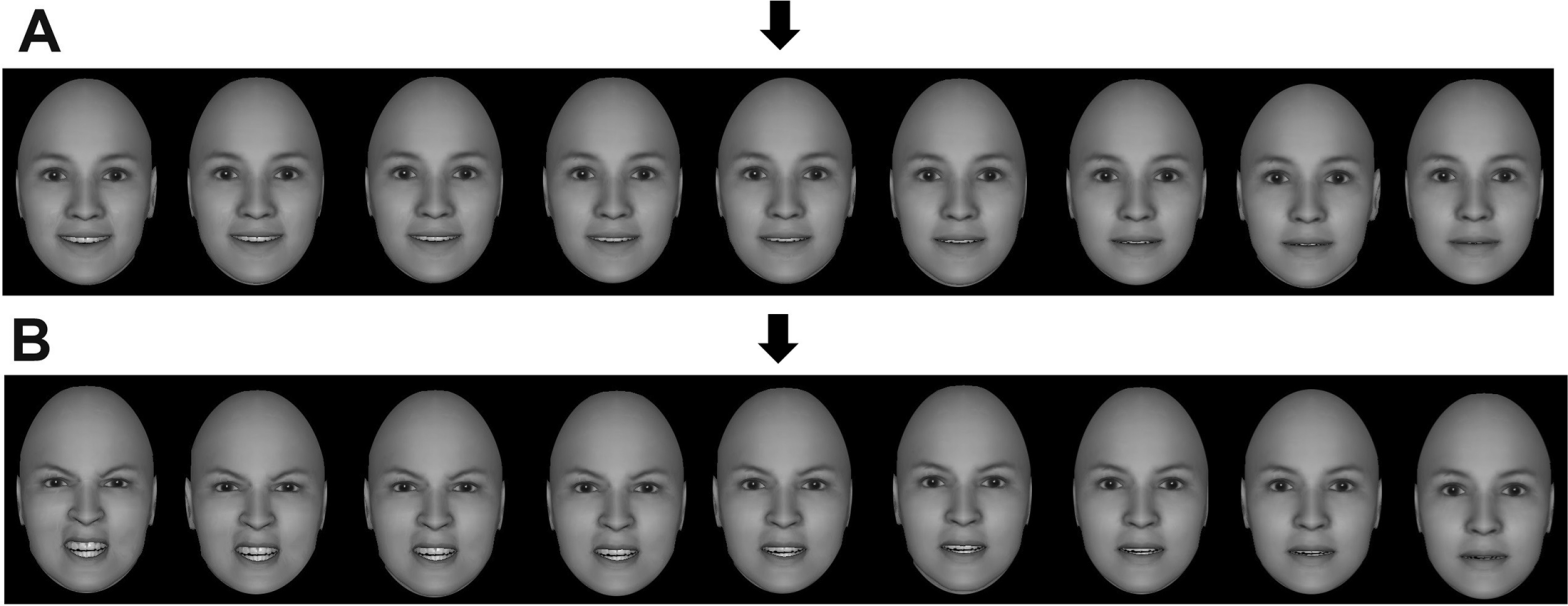
(A) Increasing levels of facial happiness. (B) Increasing levels of facial anger. The middle face in each row (highlighted with an arrow) served as a standard for comparison.

The stimuli were two-dimensional (2-D) gray images of artificial faces without hair or other external features. The faces measured approximately 5.5 cm × 4 cm. From a distance of 50 cm, the faces subtended a visual angle 6.3° vertically and 4.6° horizontally. These were created with Singular Inversions FaceGen Modeller 3.2 (Inversions, 2008). The FaceGen software deploys a three-dimensional (3-D) morphable model of faces and has been used extensively in the literature to generate artificial faces (Fitousi, 2021a, Fitousi, 2021b, Fitousi, 2020). One of its great advantages is that it allows researchers to control for the level of various facial dimensions (e.g., age, emotion) in a parametric fashion (Blanz & Vetter, 1999). To create the emotional face ensembles in this experiment, I first generated a single front view of a young Caucasian identity. I then changed the desired parameters of happiness and anger by moving two corresponding sliders across nine equally distanced steps on each emotion, starting from the lowest value possible to the highest. The resulting faces can be seen in Figure 1, where the nine levels of emotion for happy (top panel) and angry (bottom panel) faces are presented. The angle, lighting, and other perceptual parameters of the faces were held fixed. The “Sync Lock” option was checked to afford synchronized contributions of texture and shape. The faces were numbered on a 1 to 9 scale according to the strength of emotional expression they conveyed. The face with the highest level of emotional expression received the value of 9. This numbering system afforded the construction of an “emotion scale” in order to produce face ensembles with a given average.

**Figure 2.**
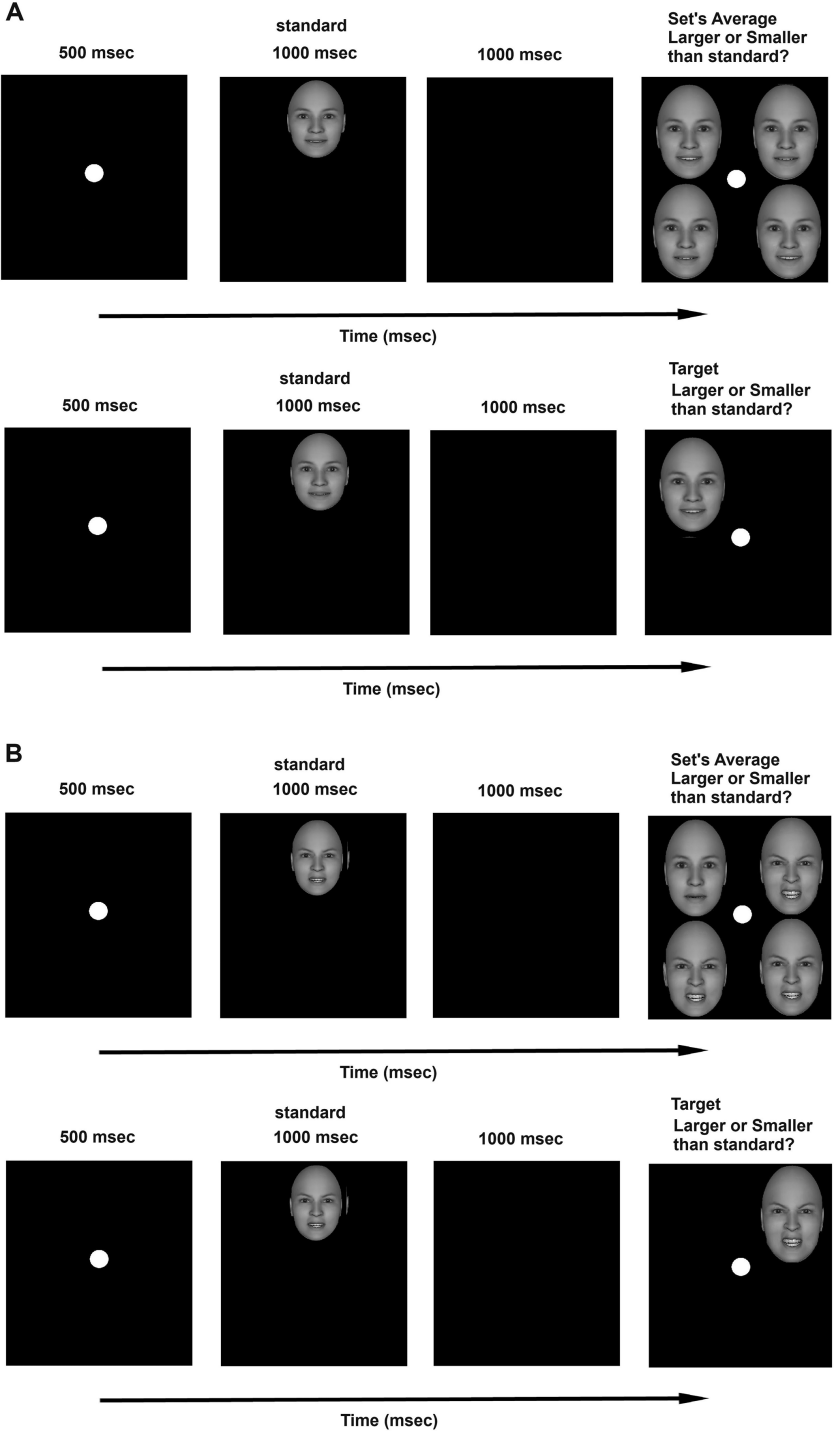
Experiment 1: The time course of a typical trial in the experiment. (**A**). Examples of redundant and single-target conditions with happy faces. (**B**). Examples of redundant and single-target conditions with angry faces.

Face-ensemble displays consisted of four faces, one on each spatial quadrant (see Figure 2). Each display presented variable degrees of emotional expression (e.g., anger) and had a known average that was either larger or smaller than 5. Single-target displays consisted of a single face, appearing in one of four possible quadrants. The facial expression of this face could receive any one of the values on the range 1 to 9 except that of the standard, which was 5. Emotion (angry, happy) was tested across participants. There were 16 unique ensemble displays (see Figure 2), with the following averages of emotion: 1.5, 1.75, 2.5, 3.25, 3.5 3.75, 4.5, 4.75, 5.5, 5.75, 6.0, 6.25, 6.5, 7.5, 8, and 8.5. So, for example, a face display with an average anger of 6.0 was created by placing angry faces 8, 7, 6, and 3 in the display. Each set was build to represent a given average, and in most cases, there were no repetitions of values in the set. Half of the displays had an average below 5.0, while the other half had an average above 5.0. The total average of all displays was 5.0. The faces on each display were arranged relative to a white fixation point (1.5 cm diameter, which is 1.71°) at the top-left, top-right, bottom-left, and bottom-right corners (see Figure 2). The edge-to-edge horizontal and vertical distances between neighboring faces amounted to 6 cm (= 6.86°). The four images occupied an area of 12 cm × 12 cm (= 13.68°).

Each experimental block consisted of 128 trials. Half of the trials (64) were ensemble displays, while the other half were single-target displays. In the ensemble displays, all 16 possible averages mentioned earlier were presented equally often. In the single-target displays, all eight possible emotion levels created were presented with the same frequency and could appear equally often, at one of the four quadrants of the screen. The type of emotion (happy or angry) was manipulated across observers. Each observer performed in 12 such blocks. In total, each observer completed 1,536 trials.

#### Procedure and design

Figure 2 illustrates the time course of a typical trial. Each trial started with a fixation point for 500 ms, and then the average face (“standard”) appeared on the screen for 1,000 msec, disappeared, and the target display appeared until response. The target display could be either a four-face ensemble or a single-target face. The standard was presented always at a fixed location at the top center of the screen for 1,000 ms and then disappeared before the target-display presentation. The standard face was smaller in size than the target faces (5.5 × 4.5 cm). The participant’s task was to indicate whether the display’s average emotional expression (whether it was an ensemble or a single face) was higher or lower than that of the standard. The standard was fixed and represented the average of all displays and faces in the experiment, which was 5. Participants pressed a right-hand key if the average of the display (or single target) was larger than the standard, and a left-hand key if the average of the display (or single target) was smaller than the standard. Both speed and accuracy were highlighted. Of the total 50 participants, 27 were allocated to a version of the experiment with happy faces and 23 to a version with angry faces.

### Results

Data were analyzed using R statistical software (R Core Team, 2017). The capacity analyses were performed with the sft package (Houpt, Blaha, McIntire, Havig, & Townsend, 2014). RTs slower than 5,800 ms or faster than 150 ms were removed from analysis. The data of two observers were removed because they did not comply with the required error rate (less than 30%). One belonged to the happy face group and one to the angry face group.

#### Averaging

**Figure 3.**
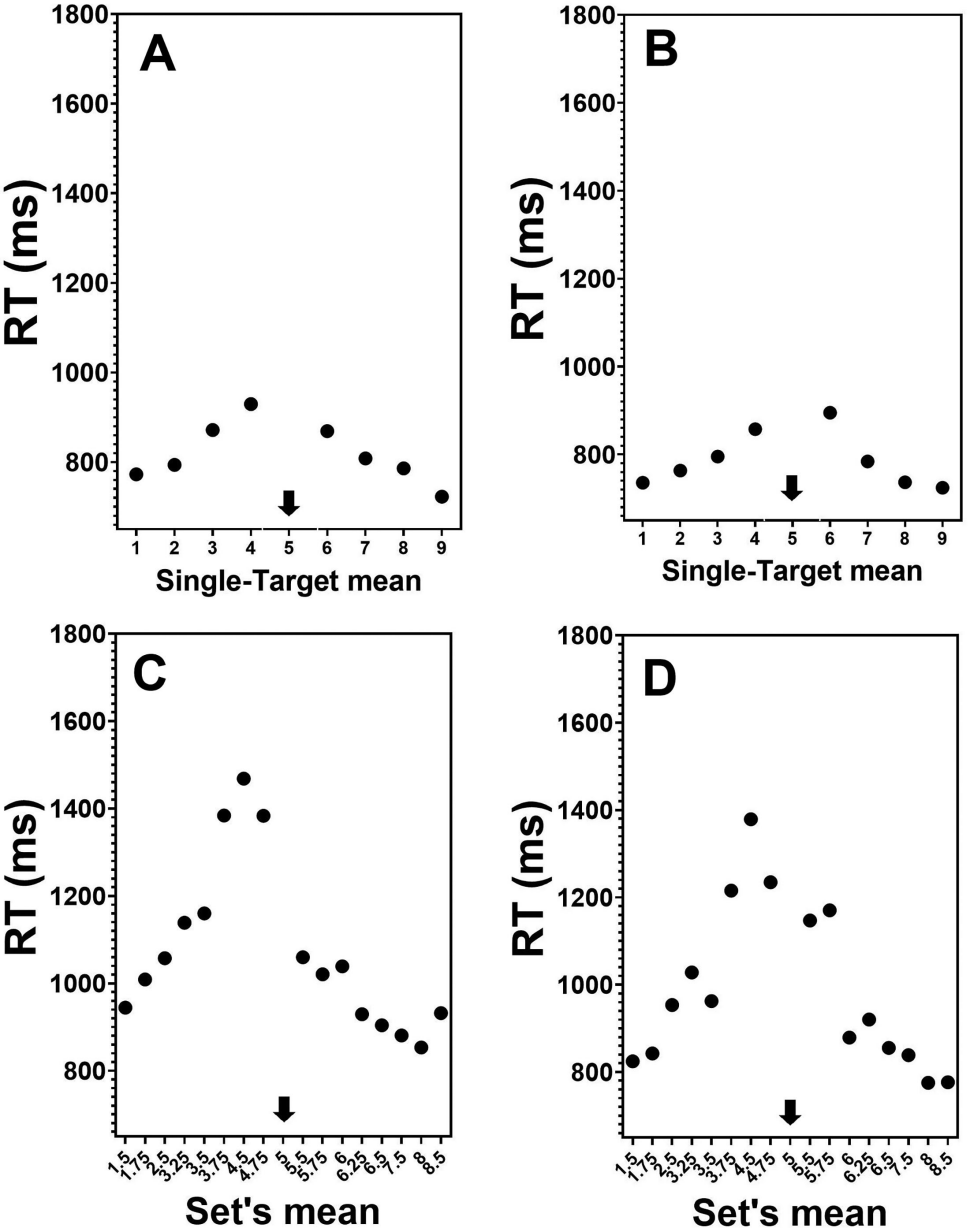
Mean RTs (ms) as a function of judged set’s average in comparison to a standard of 5 (in arbitrary units of emotion). The standard value is marked by a black arrow. (A) Single-target condition happy faces. (B) Single-target condition angry faces. (C) Ensemble condition happy faces. (D) Ensemble condition angry faces.

Figure 3 presents mean RTs (across participants) as a function of the judged set’s mean, separately for single-target and four-target (ensemble) displays. As can be noted, the patterns for single-target and four-target displays are comparable for both happy and angry faces. In all conditions, RTs decreased monotonically as the distance between the display’s average and the standard increased. This is a classic finding in psychophysics (Dehaene, Dupoux, & Mehler, 1990; Fitousi & Algom, 2020) that is often attributed to the graded discriminability of stimuli.

**Figure 4.**
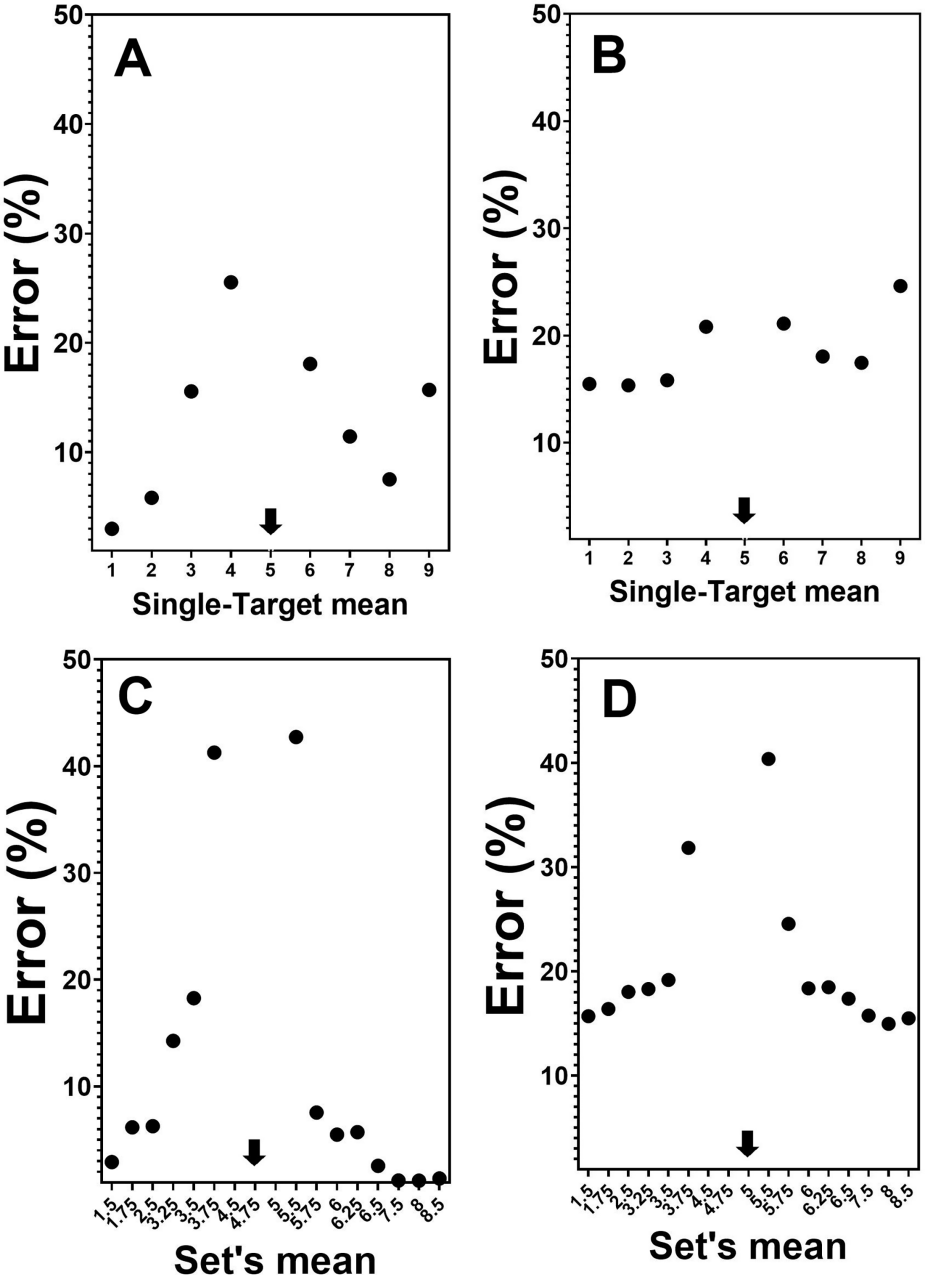
Error rates (%) as a function of judged set’s average in comparison to a standard of 5 (in arbitrary units of emotion). The standard value is marked by a black arrow. (A) Single-target condition happy faces. (B) Single-target condition angry faces. (C) Ensemble condition happy faces. (D) Ensemble condition angry faces.

One point to note is that the psychophysical function relating RTs to sample-test distance looks shifted to the left from where the peak RT is expected (i.e., the actual reference face). This can reflect the so-called amplification effect when the average feature is overestimated due to biased sampling of more salient items (Kanaya, Hayashi, & Whitney, 2018; Iakovlev & Utochkin, 2021). In case of faces, more intense facial expressions are likely to be amplified (Goldenberg, Weisz, Sweeny, Cikara, & Gross, 2021). Interestingly, this bias is less salient in the error data presented in Figure 4. The psychophysical function is similar to that observed with RTs. Errors decrease as the distance from the mean increases. For some reason, the highest item in the single-target displays in the error data exhibited a slight deviation from the level of error expected. This anomaly does not occur in the RT data, and I have no ready explanation for it.

The resemblance between the single-target and multiple-target (ensemble) RT patterns strengthens the idea that these were subjected to the same cognitive operation. This conclusion makes sense also when considered from a mathematical perspective. Note that the averaging operation is independent of the number items (as long as it is greater than 0) and can be applied even to a single item.^1^ The upshot is that the brain extracts a summary value in the same fashion, whether a single item or many items are presented, and is doing so in a similar way. This gives currency to the deployment of single-face displays in this and the next experiment. The single-target condition is a necessary condition for the application of the redundant-target methodology and the capacity coefficient.

#### Redundancy gains/losses

**Figure 5.**
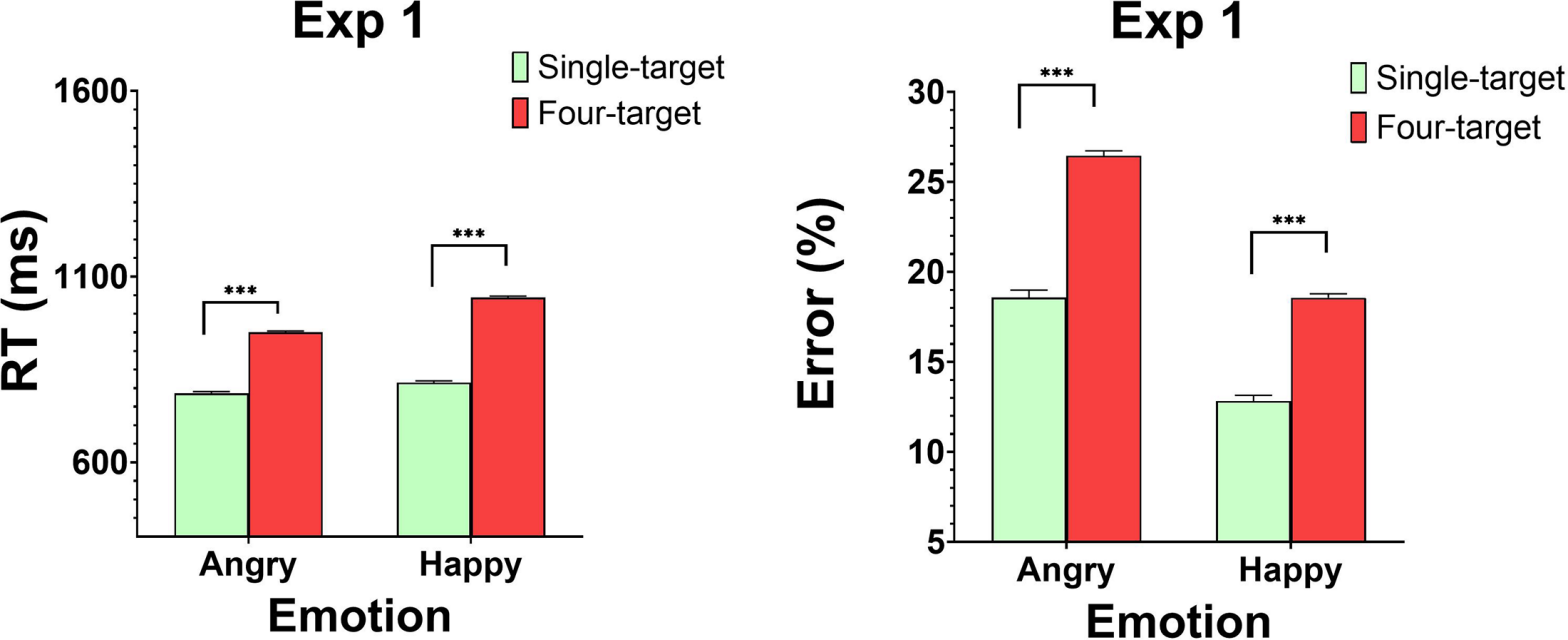
Experiment 1: Mean RTs (left) and mean error rates (right) as a function of emotion (angry, happy) and display type (single target, ensemble). * *p* < 0.05, ** *p* < 0.01, *** *p* < 0.0001.

Mean RTs in single- and four-face target displays were compared (see Figure 5). Observers who performed with happy faces exhibited a redundancy loss, namely, slower mean RTs in the four-face displays compared to the single-face displays, *t*(26) = −8.01, *p* < 0.005. Comparable redundancy losses were documented with error rates, such that more errors were made with four-face displays compared to single-face displays, *t*(26) = −6.20, *p* < 0.005. Observers who performed with angry faces exhibited comparable results. Redundancy losses were documented for RTs, *t*(22) = −8.28, *p* < 0.005, and error rates, *t*(22) = −9.14, *p* < 0.005. These results suggest that adding more faces to the ensemble hampers rather than facilitates performance. However, as demonstrated in the capacity analysis and in Appendix B, this may not necessarily dictate limited-capacity processing.

#### Capacity coefficient

**Figure 6.**
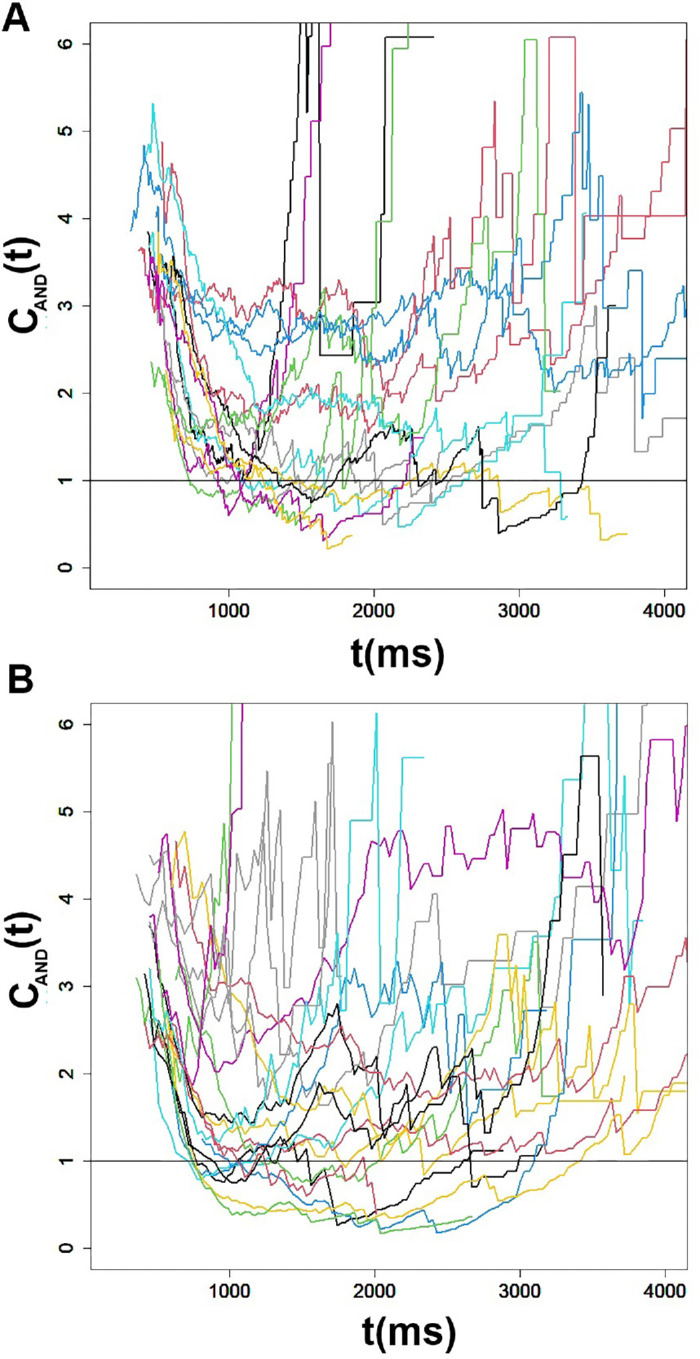
Experiment 1: The capacity coefficient of the AND type *C*_*AND*_(*t*) for each participant. The line at *C*_*AND*_(*t*) = 1 is diagnostic, since *C*_*AND*_(*t*) > 1 entails super-capacity, *C*_*AND*_(*t*) = 1 signals unlimited capacity, and *C*_*AND*_(*t*) = 1 points to unlimited capacity. All participants exhibited *C*_*AND*_(*t*) > 1 for most of *t* supported by dedicated statistical tests. (A) Angry faces. (B) Smiling faces.

The conjunctive AND capacity coefficient *C*_*and*_(*t*) was computed for each observer according to Equation 7. Figures 6A and 6B present the values of the capacity coefficient for observers who performed with angry and happy face displays, respectively. As can be noted, all observers exhibited values of the capacity coefficient that were above 1 for most of *t*. This was tested statistically using the statistics developed by Houpt and Townsend (2012) and implemented with the sft R package (Houpt et al., 2014) at the individual level.

The dedicated statistics (*z*-score) (Houpt & Townsend, 2010) developed to assess the statistical significance of the capacity coefficient was computed for each observer. The null hypothesis was that the observer performed according to the unlimited-capacity parallel model (UCIP), which predicts *C*_*and*_ = 1 (Houpt, Townsend, & Donkin, 2014). The test is two-sided. All observers exhibited positive and significant values of the statistics (all *p* < 0.05). These results imply that *C*_*and*_ > 1; namely, performance was super-capacity for all observers. These results suggest that averaging is a highly efficient process, in the sense that adding more faces to the ensemble facilitates rather hinders performance. The capacity coefficient compares the efficiency of the ensemble processing to the expected efficiency based on the processing of the individual faces in a parallel, exhaustive unlimited-capacity system. An AND capacity larger than 1 (*C*_*and*_ > 1) therefore supports the conclusion that the efficiency of the ensemble coding exceeds that predicted by the individual faces. This finding may seem at odds with the redundancy losses documented. But it should be noted that redundancy gains/losses are based on the mean RT statistics, whereas the capacity coefficient is a theoretically driven measure that is measured on the entire RT distribution, and as such provides a more sensitive and accurate measure of performance. Moreover, in Appendix B, I outline a proof of existence that a super-capacity system can generate either redundancy gains or redundancy losses, depending on the degree of its super-capacity.

### Discussion

The results of Experiment 1 showed that (a) participants are capable of extracting the average emotion of a set of faces, a finding that replicates earlier studies (Haberman & Whitney, 2007; Son et al., 2023; Yang et al., 2013), (b) the averaging operation is slower and more error prone when more faces are added to the display, but (c) processing capacity improves rather than hindered by adding more faces to the display, and (d) happy and angry face ensembles were subjected to comparable processing mechanisms. The capacity coefficient results suggest that processing is held according to a coactive super-capacity architecture in which every face in the ensemble contributes an activation that is proportional to its distance from the standard (Utochkin, Choi, & Chong, 2023). It is consistent with the view that averaging of emotion is not only a preattentive process that does not require attention but rather a gestalt-like process, in which the sum is greater than its parts (Haberman & Whitney, 2007; Leib et al., 2014). It is interesting to compare these results to other studies that have shown that ensemble processing of more items produced faster RTs in averaging facial expressions (Cho et al., 2023) and orientations (Cha & Chong, 2018; Robitaille & Harris, 2011). In yet another study, using the simultaneous-sequential paradigm, Attarha, Moore, and Vecera (2014) have demonstrated an unlimited capacity for ensemble processing of size. These studies are essentially consistent with a super-capacity system, because they show that processing is more efficient as workload is increased. As I show in Appendix B, a super-capacity system can produce opposite patterns by which mean RTs either increase or decrease with workload, depending on the magnitude of super-capacity. Therefore, even the mean RTs patterns documented here are consistent with the conclusion that averaging is a highly efficient process very much like a gestalt, where the individual items are processed according to a summative-coactive architecture.

## Experiments 2a and 2b

The goal of Experiments 2a and 2b is to test performance in the classic target-detection task (Miller, 1982; Won & Jiang, 2013). As in Experiment 1, observers were presented with displays of either single- or four-target unfamiliar faces. However, in contrast to Experiment 1, the observers’ task was that of detection rather than averaging. Specifically, observers were asked to categorize the display as conveying either happy or angry emotion. Notably, all faces in a given display posed the same identical expression (anger or happiness) and in the same emotional intensity. Thus, decision in this experiment can be based on the processing of a single target. But the question of interest is whether the observer benefits from the redundancy in the ensemble displays. Because here observers can stop processing the display once a target is found, the effective stopping rule is self-terminating (minimum time), and consequently, the disjunctive (OR) capacity coefficient is the appropriate measure of efficiency. In this OR design, if processing is parallel unlimited capacity, increasing the number of faces in the display should result in better performance due to statistical facilitation (Raab, 1962). In addition, the role of image variability (Burton, Kramer, Ritchie, & Jenkins, 2016; Fitousi, 2024) is also tested. In Experiment 2b, image variability is induced, such that images of different identities were presented, whereas in Experiment 2a, this factor is removed, and each ensemble display consisted of four replicas of the same identity.

### Method

#### Participants

Sixty-eight participants were recruited from the participants’ pool of Ariel University (mean age = 23.4, *SD* = 4.2). All participants reported normal or corrected-to-normal vision. All participants gave their informed consent. Half of the participants were assigned to Experiment 2a and the other half to Experiment 2b. The experiments reported here received the approval of the Ethics Committee of Ariel University (AU-SOC-DF-20230205).

#### Stimuli

**Figure 7.**
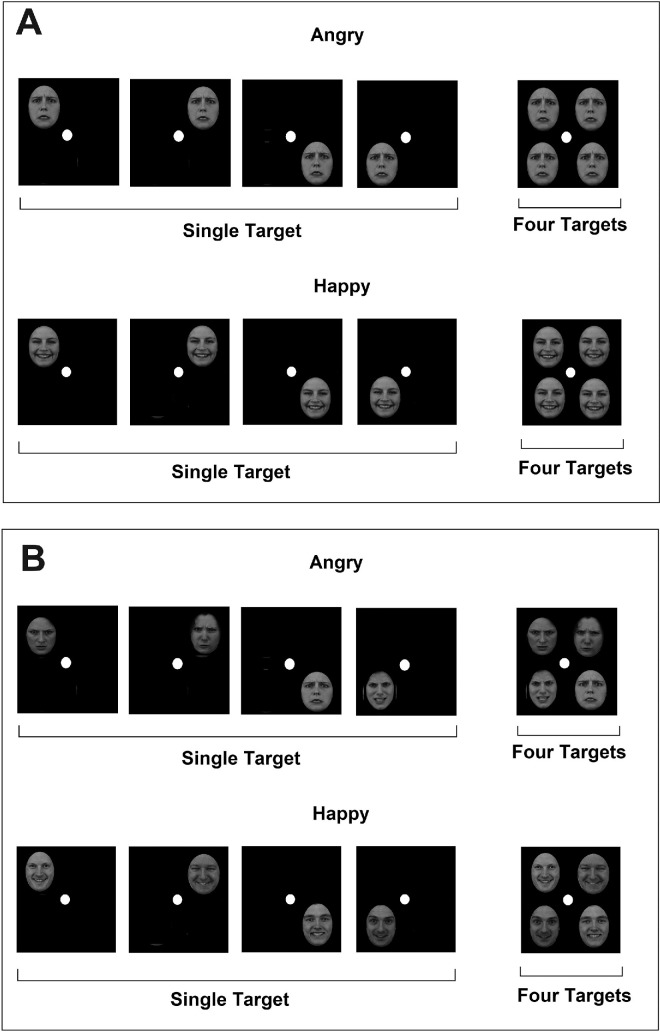
Experiments 2a and 2b: (A) Examples of single- and four-target displays of unfamiliar identities with no image variability in Experiment 2a. (B) Examples of single- and four-target displays of unfamiliar identities, including image variability.

Face stimuli were retrieved with permission from the Karolinska Directed Emotional Face (KDEF) archive (Lundqvist, Flykt, & Öhman, 1998). This archive consists of dozens of facial identities that appear as color images. The faces were photographed in frontal view while displaying various emotional expressions according to professional standards. I randomly selected eight facial identities (four males and four females) expressing anger or happiness. The images were converted to grayscale photos using the free GIMP software, and measured approximately 5.5 cm × 4 cm. Seen from a distance of 50 cm, the faces subtended a visual angle of 6.3° vertically and 4.6° horizontally. They were cut and placed in a standard oval shape. In general, the displays were comparable to those presented in Experiment 1 in terms of size and appearance. In the four-target condition, four identical replicas of the same facial identity in the same emotional expression (e.g., angry) were arranged on the display (top-left, top-right, bottom-left, and bottom-right) around a white dot (1.5 cm diameter) that served as a fixation point (see Figure 7). The horizontal and vertical edge-to-edge distances between neighboring images amounted to 6 cm. The four images occupied an area of 12 cm × 12 cm. In the single-target condition, a single image appeared in one of the four possible locations (top-left, top-right, bottom-left, or bottom-right). Thus, there were four possible displays (see Figure 7). In total, there were 16 four-target unique displays (8 identities × 2 emotions) and 64 single-target unique displays. These were created by presenting one of the images at one of the four quadrants. Multiple-face displays always presented the same gender. Experiments 2a and 2b differed only with respect to image variability, such that face images in a multiple-face display were either replicas of the same image, and therefore the same identity (Experiment 2a), or were different images, and therefore different identities (Experiment 2b). Identities in each display were of the same gender and presented with equal frequency across all displays.

#### Design and procedure

Design and procedure for Experiments 2a and 2b were identical. In each experimental block, the number of single-target and four-target trials was equated by presenting each of the possible 16 four-target displays four times (4 × 16 = 64), while single-target displays were presented only once (1 × 64 = 64). Thus, in total, each block consisted of 128 trials, of which half (64) were single targets and half (64) were four-target displays. In this way, the number of happy and angry displays was also equated. Thus, the design encapsulated the redundant-target critical trials for happy and angry displays. Each participant completed 24 consecutive blocks of trials in two separate days of testing. This amounted to 3,072 trials (2 × 12 × 128). This considerably large number of trials is necessary for conducting analyses on RT distributions, as is the case with the capacity coefficient. Each experimental session started with a short explanation and an example. Both accuracy and speed were highlighted by the experimenter. On each trial, observers were asked to judge whether the display contained a happy or an angry face(s) by pressing one of two buttons. A short break separated each block. Each trial started with presentation of a fixation point for 500 ms, then a face display was presented on the screen until the participant responded, then the screen was erased, and after 200 ms, another face display was presented on the screen. Happy and angry faces were randomly mapped to two response keys “M” and “Z.” RTs were recorded with an accuracy of 1 ms.

### Results

#### Experiment 2a

**Figure 8.**
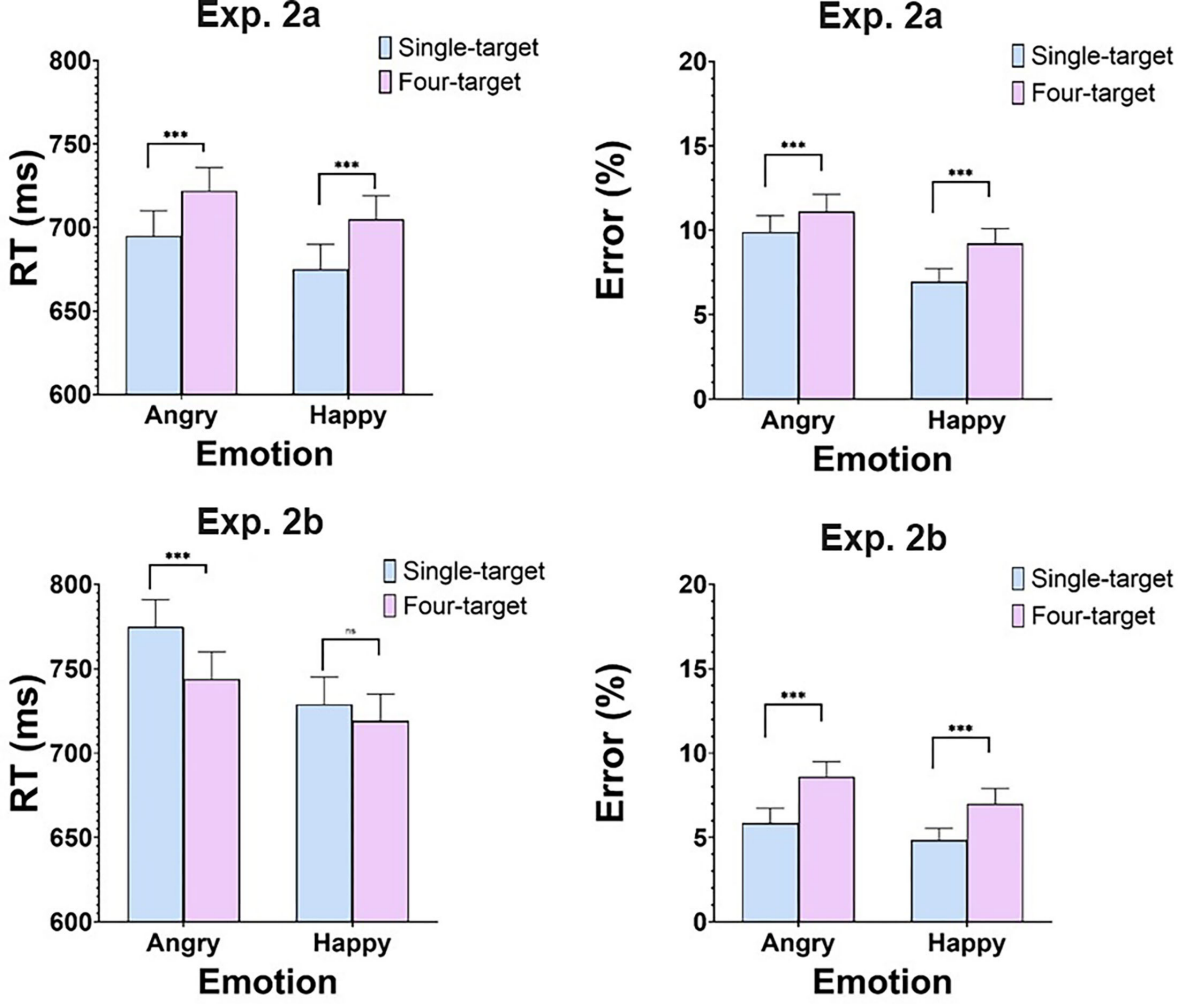
Experiments 2a and 2b: Mean RTs (left) and percentage of error rates (right). Error bars are standard error of the mean. *** *p* < 0.0001.

RTs larger than 150 ms or smaller than 2,800 ms were removed from analysis. Error rates amounted to 9.1% of the total trials. The top panel of Figure 8 gives mean RTs and error rates as a function of number of targets (one vs. four) and emotion (angry vs. happy). A two-way ANOVA with Emotion (angry, happy) × Target (one, four) showed a main effect of Emotion, *F*(1, 33) = 12.35, *MSE* = 11318, *p* < 0.005, entailing slower responses with angry (709 ms) compared to happy (691 ms) faces. Most importantly, a main effect of Target, *F*(1, 33) = 112.5, *MSE* = 27,556, *p* < 0.0001, underscored a significant redundancy loss, such that, on average, performance was 28 ms slower with four-target displays than with single-target displays. The interaction of Emotion and Target was not significant, *F*<1. Comparable analyses on error rates mimicked the RT results. A main effect of Emotion, *F*(1, 33) = 14.65, *MSE* = 0.01, *p* < 0.0001, showed that angry faces elicited more errors than happy faces. Most importantly, a main effect of Target, *F*(1, 33) = 59.75, *MSE* = 0.01, *p* < 0.0001, documented redundancy loss in error rates too. Participants committed more errors with four-target displays than with single-target displays. The interaction of Emotion and Target was also significant, *F*(1, 33) = 4.85, *MSE* = 0.001, *p* < 0.05, reflecting larger redundancy losses for happy faces, *t*(33) = 6.46, *p* < 0.0001, than for angry faces, *t*(33) = 4.01, *p* < 0.001.

#### Experiment 2b

The same exclusion criteria on RTs as in Experiment 2a led to removal of 7.6% of the data. The bottom panel of Figure 8 gives mean RTs and error rates in this experiment. A two-way ANOVA with Emotion (angry, happy) × Target (one, four) showed a main effect of Emotion, *F*(1, 33) = 45.22, *MSE* = 43658, *p* < 0.0001, which replicated the finding from Experiment 2a, underscoring slower processing of angry than happy face-displays. Most importantly, a significant main effect of Target, *F*(1, 33) = 8.13, *MSE* = 14258, *p* < 0.005, modulated by Emotion, *F*(1, 33) = 24.52, *MSE* = 3838, *p* < 0.0001, indicated the presence of a 31-ms redundancy gain for angry face displays, *t*(33) = 3.71, *p* < 0.001, but no such effect for happy faces, *t*(33) = 1.51, *p* > 0.05. Similar analyses on error rates revealed a main effect of Target, *F*(33) = 66.32, *MSE* = 0.020, *p* < 0.0001, that was not modulated by Emotion, *F*(33) = 1.78, *MSE* = 0.0002, *p* = 0.19. In contrast to the RT data, this main effect recorded substantial redundancy losses, such that overall, error rates were higher with multiple-face displays than with single-face displays, irrespective of emotion.

Experiments 2a and 2b differed only with respect to the presence of image variability. While Experiment 2a provided clear evidence for redundancy losses for both speed and accuracy, Experiment 2b showed redundancy gains for RTs only with angry faces and substantial redundancy losses for accuracy. Taken together, these results generally point to a limited-capacity process. Recall that redundancy losses found with identical-image displays can indicate a serial or parallel system with negative (inhibitory) interactions among its channels, thus supporting a suppression model (Desimone & Duncan, 1995). In contrast, the redundancy gain found for angry faces in Experiment 2b, may support a horserace or a coactivation model (Miller, 1982). However, there is computational (Townsend & Nozawa, 1997) and empirical (Fitousi & Algom, 2018) evidence that redundancy gains can be still generated by a serial limited-capacity system, and redundancy losses can be generated by a super-capacity system (see Appendix B). Thus, the ultimate arbiter to decide between these candidate models should be the capacity coefficient to which I turn now.

#### The capacity coefficient

**Figure 9.**
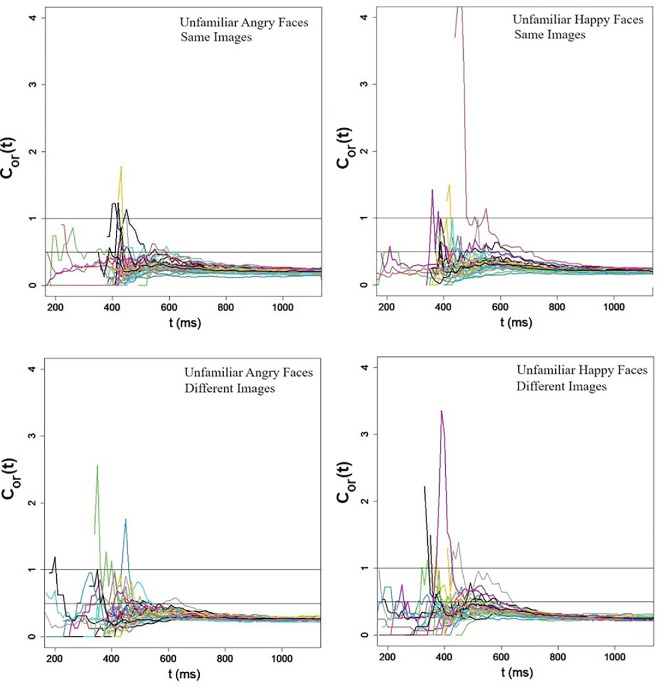
Experiments 2a and 2b: Individual-by-individual capacity coefficient *C*_*or*_(*t*) for angry and happy faces in Experiment 1a (top) and Experiment 1b (bottom). The line drawn at *C*_*or*_(*t*) = 1 is diagnostic for limited-capacity *C*_*or*_(*t*) < 1. The line drawn at *C*_*or*_(*t*) = 1 is diagnostic for extremely limited-capacity *C*_*or*_(*t*) ≪ 1.

Capacity analyses were held using the statistics for the disjunctive (OR) capacity coefficient developed by Houpt and Townsend (2012) and implemented with the sft R package (Houpt et al., 2014) at the individual level. The capacity coefficient was computed separately for happy and angry face displays, experiments, and participants. Figure 9 presents these capacity coefficient functions. One can readily note that the capacity coefficient in all cases and for all observers was below the critical value of 1 along the entire time range, irrespective of emotional expression or image variability. This result strongly indicates limited capacity. Moreover, a closer look at these values reveals that the capacity coefficient values were mostly lower than 0.5, a value that indicates extremely limited capacity (Townsend & Wenger, 2004b). This comes as a great surprise after the finding of super-capacity in the averaging task in Experiment 1. Statistical tests (Houpt & Townsend, 2010) were performed separately for each participant, condition, and experiment. These revealed that the capacity coefficient values were significantly lower than those of a benchmark UCIP (unlimited-capacity independent parallel) model for all participants (all *Z*s < −41, *p* < 0.00001). These results were robust across participants, conditions, and types of displays and thus provide strong evidence for a serial or parallel architecture with negative (inhibitory) interactions among the faces in the display. These results refute a coactive system with positive (faciliatory) activations.

### Discussion

The empirical patterns recorded in Experiments 2a and 2b converged on the same theoretical conclusion, that is, ensemble processing of emotional faces in the redundant-target task is an extremely limited-capacity process, irrespective of emotional expression and image variability. First, except in one case (angry faces with image variability), redundancy losses, rather than gains, were documented in both RTs and accuracy rates. Second, capacity coefficient values were consistently lower than 1 (and mostly smaller than 0.5), indicating extremely limited-capacity processing (Townsend & Wenger, 2004b). These results are in marked contrast to the super-capacity found in the averaging task of Experiment 1. They suggest that averaging and redundant-target detection are governed by different mechanisms. The averaging task results support a coactive system, or a parallel system with positive interactions among channels (faces), whereas the redundant-target results are consistent with a serial or parallel system with negatively correlated channels. These architectures are consistent with a suppression model (Desimone & Duncan, 1995), according to which competition between items in the visual field results in mutual inhibition. They are also in line with (Fitousi, 2021c) recent findings with emotionally neutral faces.

These results may suggest that redundant-target and averaging tasks are held differently. Averaging is an automatic preattentive process, whereas redundant-target detection is an attention-demanding process. These redundant-target results are generally inconsistent with those reported by Won and Jiang (2013), who found redundancy gains rather than redundancy losses. However, note that redundancy gain/losses provide weaker evidence for capacity than the capacity coefficient because (a) they are based on mean RTs and not on entire RT distributions, and (b) unlike the capacity coefficient, which is a theory-based measure, their interpretation is not unambiguous (Townsend & Nozawa, 1997).

The finding of extremely limited capacity with unfamiliar faces is quite surprising given the super-capacity observed in the averaging task. Previous research (Awad, Emery, & Mareschal, 2023) has documented important influences of familiarity on the processing of face ensembles. Thus, the present results certainly invite replication and generalization with familiar faces.

## Experiment 3a and 3b

The goal of Experiments 3a and 3b is to test whether the extremely limited-processing capacity observed in Experiments 2a and 2b also generalizes to ensembles of familiar faces. Many researchers believe that familiar and unfamiliar faces are processed in qualitatively different ways (Bruce, Henderson, Newman, & Burton, 2001; Burton, Schweinberger, Jenkins, & Kaufmann, 2015). Familiar faces are handled at a semantic level, whereas unfamiliar faces are treated at the image level (Fitousi & Azizi, 2023; Fitousi, 2024). This critical difference may be responsible for the robust finding that performance with familiar faces is often faster and more accurate than with unfamiliar faces. Moreover, it has been shown that familiar and unfamiliar faces activate separate brain loci (Natu & O’Toole, 2011). The impact of familiarity on the processing of face ensembles has been recently investigated (Awad, Emery, & Mareschal, 2023). These researchers have shown that when a familiar face appeared within an ensemble of faces, perception was biased toward this face’s emotion, regardless of its intensity. However, when all faces were unfamiliar, the presence of any high-intensity emotional face biased ensemble perception toward its emotion. This suggests that ensembles with familiar and unfamiliar faces may be subjected to different capacity allocation strategies, a conjecture that will be tested here with the capacity coefficient. To this end, I have deployed the same methodology as in Experiments 2a and 2b, with images of two famous Israeli politicians.

### Method

#### Participant

A new sample of 58 participants (mean age = 22.3, *SD* = 2.2) who did not take part in previous experiments was recruited from the participants pool of Ariel University. Twenty-four were assigned to Experiment 2a and 34 to Experiment 2b. These experiments received the approval of the Ethical Committee of Ariel University (AU-SOC-DF-20230205).

#### Stimuli

Face images with frontal views of two famous Israeli politicians, Binyamin Netanyahu and Yair Lapid, were retrieved from Google’s photo search engine. The faces conveyed either happy or angry emotional expressions. Four different images were selected for each facial identity (two of them conveyed anger and two happiness). In total, there were eight different photos. The images were converted to grayscale photos using the free GIMP software. In the four-target condition, four identical images of the same politician (e.g., Yair Lapid) displaying the same emotional expression (e.g., anger) were presented on the display (top-left, top-right, bottom-left, and bottom-right) around a white dot (1.5 cm diameter) that served as a fixation point. In total, there were 16 four-target unique displays (2 identities × 4 images × 2 emotions). In addition, I created 64 single-target unique displays by presenting, in each display, only one of the images at one of the four quadrants of the screen. Experiments 3a and 3b differed with respect to the presence of image variability. The multiple-face displays in Experiment 3a consisted of four replicas of the same image, whereas the multiple-face displays in Experiment 3b incorporated different images of the same identity and the same emotional expression.

#### Design and procedure

These were identical to those reported in Experiments 2a and 2b.

### Results

#### Experiment 3a

**Figure 10.**
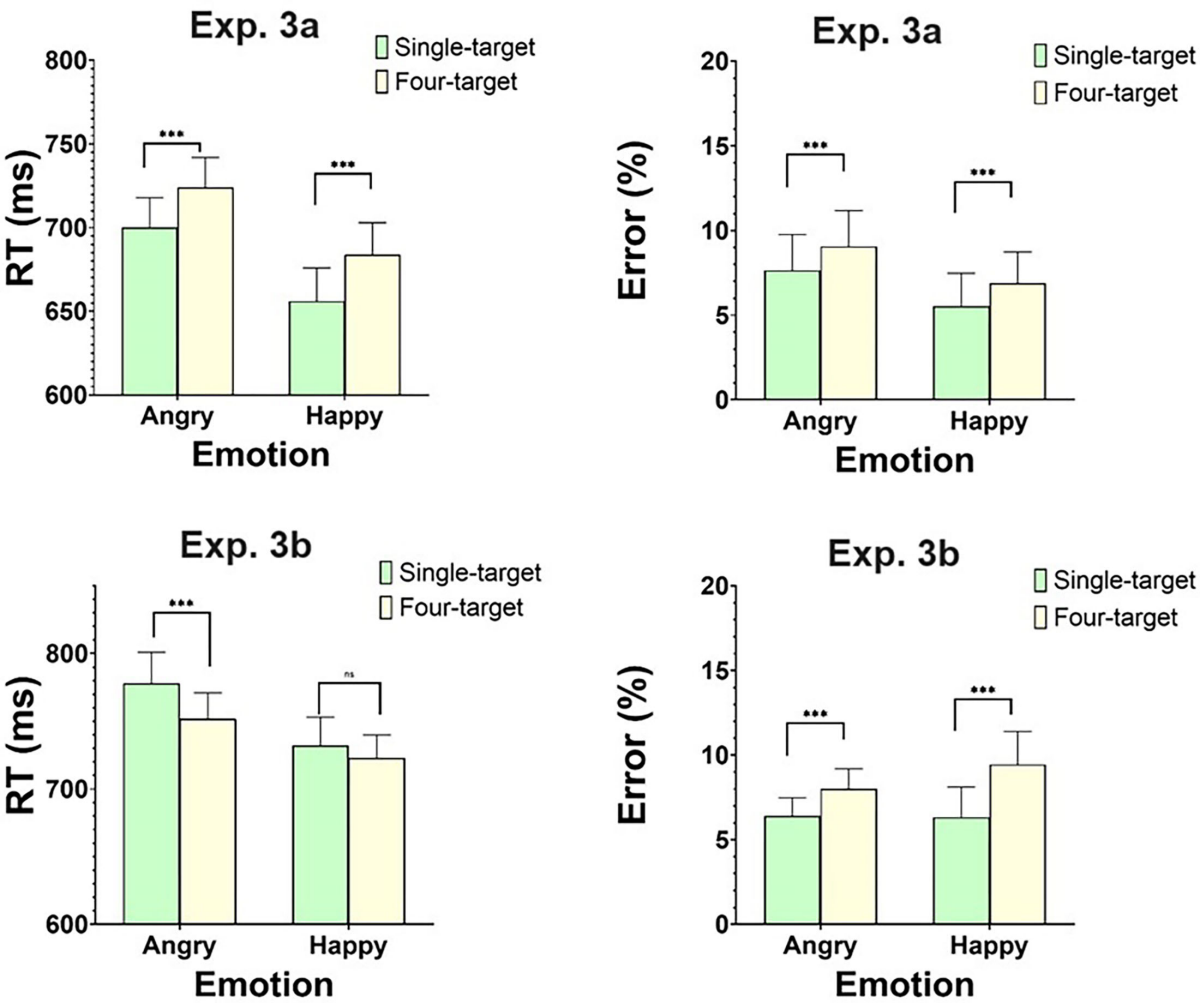
Experiments 3a and 3b: Mean RTs (left panels) and percentage of Error rates (right panels). Error bars are standard error of the mean. *** = *p* < 0.0001.

RTs larger than 150 ms or smaller than 2,800 ms and error trials were excluded. These amounted to 7.47% of the total trials. Separate analyses for happy and angry displays were performed to assess the presence of redundancy gains. The top panel of Figure 10 gives mean RTs and error rates in these conditions. Longer RTs in four targets compared to single-target trials were documented. These redundancy losses (rather than redundancy gains) replicated the ones we found in Experiment 2a with unfamiliar faces. This result was corroborated by a two-way Emotion × Target ANOVA, which revealed a main effect of Target, *F*(1, 23) = 119.7, *MSE* = 16,591, *p* < 0.0001, such that four-target displays were responded to 26 ms slower than single-target displays. The effect of Emotion was also significant, *F*(1, 23) = 32.9, *MSE* = 40,931, *p* < 0.0001, entailing slower responses with angry (712 ms) compared to happy (671 ms) faces. The interaction of Emotion and Target was not significant (*F* < 1). Comparable analyses on error rates exhibited similar results to those observed with RTs (see right panel of Figure 10). A main effect of Target, *F*(1, 23) = 31.59, *MSE* = 0.004, *p* < 0.0001, which was not modulated (*F* < 1) by Emotion, confirmed that participants made more errors with four-target displays than with single-target displays. The main effect of Emotion was not significant, *F*(1, 23) = 3.64, *MSE* = 0.01, *p* = 0.06. These results provide a full replication of the results with unfamiliar faces in Experiment 2a.

#### Experiment 3b

The same trial exclusion criteria as in previous experiments led to the removal of 9.2% of the data. The bottom panel of Figure 10 gives mean RTs and error rates in the experiment. A two-way ANOVA with Emotion (angry, happy) × Target (one, four) exhibited a main effect of Emotion, *F*(1, 33) = 32.02, *MSE* = 48,239, *p* < 0.0001, with angry faces being processed less efficiently than happy faces. The effect of Task, *F*(1, 33) = 7.08, *MSE* = 10,401, *p* < 0.05, which was modulated by Emotion, *F*(1, 33) = 7.40, *MSE* = 2,413, *p* < 0.05, revealed a redundancy gain for angry faces, *t*(33) = 3.01, *p* < 0.05, but not for happy faces, *t*(33) = 1.61, *p* > 0.05. This is the exact pattern observed in parallel Experiment 2b. Comparable analyses on error rates revealed an effect of Target, *F*(1, 33) = 66.88, *MSE* = 0.019, *P* < 0.0001, which was modulated by Emotion, *F*(1, 33) = 7.42, *MSE* = 0.001, *p* < 0.05. In contrast to the RT results, this effect on error rates pointed to the presence of redundancy losses that were larger with happy, *t*(33) = 6.32, *p* < 0.0001, than with angry, *t*(33) = 5.74, *p* < 0.0001, faces. These results offer a full replication of those obtained with familiar faces in Experiment 2b.

Taken together, the results of Experiments 3a and 3b replicated the exact patterns of Experiments 2a and 2b, which were held with familiar faces. When no image variability was present, redundancy losses surfaced with both angry and happy faces and for both RTs and error rates. When displays induced image-variability, a redundancy gain was observed for RTs with angry faces only, but redundancy losses for errors resurfaced with both angry and happy faces.

#### The capacity coefficient

**Figure 11.**
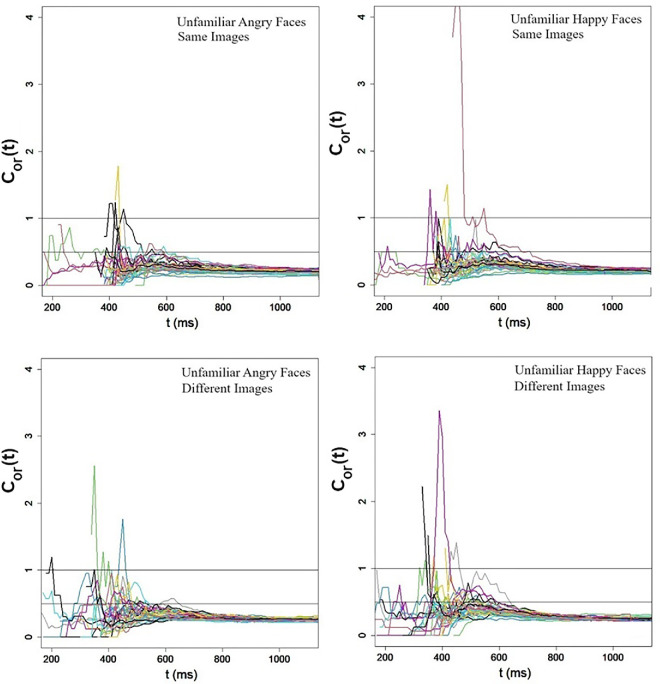
Experiments 3a and 3b: Individual-by-individual capacity coefficients for angry and happy faces in Experiment 1a (top) and Experiment 1b (bottom). The line drawn at *C*_*or*_(*t*) = 1 is diagnostic for limited-capacity *C*_*or*_(*t*) < 1. The line drawn at *C*_*or*_(*t*) = 1 is diagnostic for extremely limited-capacity *C*_*or*_(*t*) ≪ 1.

The capacity coefficient was computed separately for happy and angry face displays in an individual-by-individual fashion. The results replicated those found in Experiments 2a and 2b. As can be noted in Figure 11, the capacity coefficient dwells below 0.5 along the entire time range, entailing extremely limited processing capacity for both angry and happy emotional expressions, irrespective of the presence or absence of image variability. Dedicated statistical tests (Houpt & Townsend, 2012) performed separately on the data of each participant confirmed this observation. In all cases, the capacity coefficient values were significantly lower than those of a benchmark UCIP (unlimited-capacity independent parallel) model (all *Z* values ranged between −48.39 and *Z* = −40.61, *p* < 0.00001). These results fully replicate the findings from Experiments 2a and 2b. They provide strong evidence that the redundant-target task with ensemble faces is extremely limited capacity.

### Discussion

Experiments 3a and 3b replicated the findings from Experiments 2a and 2b with ensembles of familiar faces. The results pointed to extremely limited capacity in the redundant-target task, an outcome that can be generated by either a serial or parallel architecture with negative interactions. This in contrast to the averaging task, which is characterized by super-capacity.

## General discussion

The present study tested the capacity and architectural characteristics of two apparently opposite tasks. The first task is averaging of the emotional expression of face ensemble, in which the observer should ignore the individual faces and extract the gist of the display. The second is a redundant-target task, in which the observer can make a response on the basis of a single face and thus ignore the overall context. The present results tell a clear story. The averaging can be characterized as a super-capacity process, one that benefits from increasing the number of faces in the display, while the redundant-target task is an extremely limited process, one that is compromised by increasing workload. These two contrast outcomes suggest that, although dealing with the same or similar ensembles, the averaging operation and the target-detection operation are sustained by different processing architectures. Averaging is likely governed by a coactive system, or a parallel system with positive interactions, whereas target detection is sustained by a serial or parallel system with negatively correlated channels. These marked differences between the two tasks also imply contrasting attentional demands. Averaging is an automatic, preattentive, and efficient process, whereas redundant-target detection is a controlled, attention-demanding, and not efficient process.

The role of attentional resources allocated to individual objects and to ensembles has been recently modeled by Baek and Chong (2020). In their model, attentional mechanisms, such as the zoom lens model (distributed attention) and a spotlight model (focused attention), were incorporated, along with early and late noise mechanisms, into the averaging process. Baek and Chong (2020) found that distributed attention led to better averaging than focused attention. It might be the case that the differences in processing efficiency observed here between averaging and visual-search tasks result from the deployment of different attentional mechanisms. In particular, observers may have used distributed attention in the averaging task and focused attention in the redundant-target task, which in turn led to differences in overall efficiency.

Another point that deserves a comment concerns the question of whether observers automatically extract the average emotion, even when they are not asked to do so, as in the case of the redundant-target task. If that were the case, then super-capacity should have been found in this task. The fact that this is not the case suggests that averaging might be an optional process that depends on task instructions but, once executed, is characterized by super-capacity. An opposite question can also be asked. Do observers pay attention to individual items when asked to extract a summary statistics? Consider the phenomenon of “robust averaging” (Cha & Chong, 2018; Cho et al., 2023; De Gardelle & Summerfield, 2011; Robitaille & Harris, 2011) – the tendency of observers to downweight or even completely discount items that are outlying from the mean of the distribution (Haberman & Whitney, 2010). This phenomenon may suggest that averaging does allow for attention to be directed to individual items. However, Utochkin, Choi, and Chong (2023) demonstrated that robust averaging naturally occurs during the pooling process. Thus, it is unnecessary to pay attention to outliers to reject them.

A word is in order regarding the possibility that observers in the redundant-target task could perform the task by focusing on one target. This is unlikely because the paradigm is designed to maximize uncertainty in the location of the target. Single targets appeared equally often in one of four possible quadrants, so the observer could not know in advance where the target is. Moreover, I documented substantial redundancy losses and extremely limited capacity in this task. These results refute the focusing hypothesis because if it were correct, then increasing the load from a single face to four faces should not have made a difference.

Another issue that deserves a comment concerns whether an averaging task with a single target is a valid practice. The answer to this question is threefold. First, mathematically speaking, this is an amply logical operation. In principle, there is no obstacle in applying the averaging operation $\bar{x}=\frac{\sum_{i=1}^{n}x_{i}}{n}$ to the single-item case *n* = 1. In that event, $\bar{x}=\frac{\sum_{i=1}^{1}x_{i}}{1}=x_{1}$. Second, the empirical patterns adduced in Experiment 1 clearly show that the RT patterns for comparing an ensemble of faces to a standard are comparable to those of comparing a single face to the standard. It is likely that the underlying representations and processing mechanisms for the extractions of summary statistics are similar for single- and multiple-item displays. Third, the application of the capacity coefficient and the redundancy gain measures necessitate the incorporation of a single-target condition. These measures are based on comparisons, but their ultimate theoretical resolution concerns the ensemble, not the single-target condition.

The capacity measurements deployed here are based on the central notion of *workload* and its expected influence on processing efficiency. But there are other related methodologies that address the temporal efficiency of processing of multi-item ensembles (Attarha & Moore, 2015; Attarha, Moore, & Vecera, 2016; Corbett, Utochkin, & Hochstein, 2023; Whitney & Yamanashi Leib, 2018). For example, Attarha and colleagues (Attarha, Moore, & Vecera, 2014) deployed the sequential-simultaneous paradigm to assess the processing capacity of circles with various diameters. While computation of the mean across ensembles was found to be of fixed capacity, computing the mean in a single ensemble was consistent with an unlimited-capacity processing. This result is in line with the present findings. An important goal of future research is to address the relations between the capacity methodology used here and the sequential-simultaneous paradigm of Attarha and colleagues. Other future goals are to generalize the present conclusions to ensembles of simple features such as line orientations and circle sizes. Computational work can incorporate diffusion processes at the local individual-item level to explain processing at the global level.

Finally, the capacity coefficients and the hazard functions have been applied here exclusively to RT distributions, but it would be desirable to model accuracy as well. Notably, the hazard functions are model-free quantities that do not require any assumptions regarding accuracy. One goal for future research would be that of building parametric models that take into consideration both response times and accuracy.

## Figures and Tables

**Figure 12.**
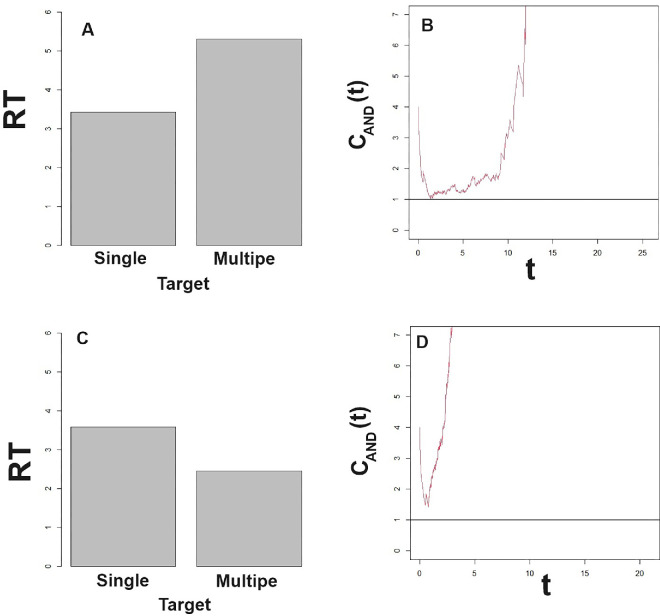
Simulations results. Intensity parameter (λ) of processing with ensemble displays was either 1.3 times larger or 2.7 larger than that with single-target displays. (A) Redundancy losses obtained with the low-intensity factor (1.3), (B) super-capacity with the low-intensity factor (1.3), (C) redundancy gains with the high-intensity factor (2.7), and (D) super-capacity with the high-intensity factor (2.7).

## Footnotes

1 Consider the averaging formula: $\bar{x}=\frac{\sum_{i=1}^{n}x_{i}}{n}$ where *n* is the number of faces in the display. This operation can be readily applied to *n* = 1. In that case, $\bar{x}=\frac{\sum_{i=1}^{1}x_{i}}{1}=x_{1}$.

## Appendix A

Estimation of the conjunctive (AND) capacity coefficient in Experiment 1 was performed by first deriving the integrated reversed hazard function *K*(*t*) (Chechile, 2011) for each of the four single-target conditions and for the ensemble (four-faces) condition. Recall that there were four types of single targets, each appearing on one of the four quadrants of the screen. The identity *K*(*t*) = ln [ *F*(*t*)]  (Townsend & Ashby, 1983; Luce, 1986) greatly assisted in computing these values. Thus, the integrated reversed hazards for the four single-target conditions are *K*_1_(*t*) = ln [ *F*_1_(*t*)] , *K*_2_(*t*) = ln [ *F*_2_(*t*)] , *K*_3_(*t*) = ln [ *F*_3_(*t*)] , and *K*_4_(*t*) = ln [ *F*_4_(*t*)] . The integrated reversed hazard function for the ensemble is given by *K*_*ens*_(*t*) = ln [ *F*_*ens*_(*t*)] . Applying Equation 7 gives the capacity coefficient:

(8) $$\begin{array}{ccc} \;C_{AND}\left(t\right) & \;= & \frac{\ln\left[\;F_{1}\left(t\right)\right]\;+\ln F_{2}\left(t\right)+\ln\left[\;F_{3}\left(t\right)\right]\;+\ln\left[\;F_{4}\left(t\right)\right]\;}{\ln[\;F_{ens}\left(t\right)]\;}\\ & \;= & \frac{\ln[\;F_{1}\left(t\right)\times F_{2}\left(t\right)\times F_{3}\left(t\right)\times F_{4}\left(t\right)]\;}{\ln[\;F_{ens}\left(t\right)]\;} \end{array}$$

Estimation of the disjunctive (OR) capacity coefficient in Experiments 2a, 2b, 3a, and 3b was held by first deriving the integrated hazard function *H*(*t*) for each of the four single-target conditions and for the ensemble condition. The well-known identity *H*(*t*) = −ln [ 1 − *F*(*t*)] = −ln [ *S*(*t*)]  facilitated computation. The integrated hazard functions for the four single-target conditions are *H*_1_(*t*) = −ln [ *S*_1_(*t*)] , *H*_2_(*t*) = −ln [ *S*_2_(*t*)] , *H*_3_(*t*) = −ln [ *S*_3_(*t*)] , and *H*_4_(*t*) = −ln [ *S*_4_(*t*)] . The integrated hazard function for the ensemble is given by *H*_*ens*_(*t*) = −ln [ *S*_*ens*_(*t*)] . Applying Equation 4 gives the capacity coefficient:

(9) $$\begin{array}{ccc} \;C_{OR}\left(t\right) & \;= & \frac{-\ln[\;S_{ens}\left(t\right)]\;}{-\ln\left[\;S_{1}\left(t\right)\right]\;-\ln S_{2}\left(t\right)-\ln\left[\;S_{3}\left(t\right)\right]\;-\ln\left[\;S_{4}\left(t\right)\right]\;}\;\\ & \;= & \frac{-\ln[\;S_{ens}\left(t\right)]\;}{-\ln[\;S_{1}\left(t\right)\times S_{2}\left(t\right)\times S_{3}\left(t\right)\times S_{4}\left(t\right)]\;} \end{array}$$

## Appendix B

The goal of these simulations is to show that either redundancy gains or redundancy losses can emerge in a super-capacity conjunctive (AND) system, depending on the level of super-capacity. To this end, I have simulated an AND system using the exponential distribution (Townsend & Ashby, 1983; Luce, 1986):

(10) $$f\left(t\right)=\left\{\begin{array}{cc} \lambda e^{-\lambda t} & t>0\\ 0 & \text{otherwise.} \end{array}\right.$$

where the parameter λ determines the intensity of processing. Larger values of λ entail more intensive processing. This is also reflected in the fact that the integrated hazard function of the exponential distribution is equal to λ, *H*(*t*) = λ (see Eq. 3.18 in Townsend & Ashby, 1983). I simulated RT distributions for four single-target displays, assuming equal intensity for all channels (targets) λ_1_ = λ_2_ = λ_3_ = λ_4_ = 0.33. A super-capacity system is characterized by intensity that is larger than the intensity of either of the single-targets (Houpt, Townsend, & Donkin, 2014; Houpt et al., 2014). To demonstrate the effect of super-capacity, I have used two λ values for the ensemble display: a low-intensity value that was 1.3 times larger than the single target’s λ and a high-intensity value that was 2.7 times larger than the single target’s λ. I then sampled 100 RTs from those distributions and computed the mean for single targets and ensemble RTs displays. I also computed the conjunctive AND capacity coefficient. As can be noted in Figure 12, when the intensity factor was low (1.3), super-capacity was documented with redundancy losses, but when the intensity factor was high (2.7), larger super-capacity was registered with redundancy gains. These results show that either redundancy gains or redundancy losses can emerge in an AND system, depending on the magnitude of super-capacity.
